# Know your tuberculosis epidemic–Is it time to add *Mycobacterium tuberculosis* immunoreactivity back into global surveillance?

**DOI:** 10.1371/journal.pgph.0001208

**Published:** 2022-10-24

**Authors:** Hannah M. Rickman, Wala Kamchedzera, Alvaro Schwalb, Mphatso D. Phiri, Morten Ruhwald, Kwame Shanaube, Peter J. Dodd, Rein M. G. J. Houben, Elizabeth L. Corbett, Peter MacPherson

**Affiliations:** 1 Clinical Research Department, London School of Hygiene & Tropical Medicine, London, United Kingdom; 2 Malawi Liverpool Wellcome Programme, Blantyre, Malawi; 3 TB Modelling Group, TB Centre, London School of Hygiene and Tropical Medicine, London, United Kingdom; 4 Department of Infectious Disease Epidemiology, London School of Hygiene and Tropical Medicine, London, United Kingdom; 5 Instituto de Medicina Tropical Alexander von Humboldt, Universidad Peruana Cayetano Heredia, Lima, Peru; 6 Department of Clinical Sciences, Liverpool School of Tropical Medicine, Liverpool, United Kingdom; 7 FIND, the Global Alliance for Diagnostics, Geneva, Switzerland; 8 Zambart, Lusaka, Zambia; 9 School of Health and Related Research, University of Sheffield, Sheffield, United Kingdom; Africa Health Research Institute, SOUTH AFRICA

## Abstract

Tuberculosis (TB) still causes 1.5 million deaths globally each year. Over recent decades, slow and uneven declines in TB incidence have resulted in a falling prevalence of TB disease, which increasingly concentrates in vulnerable populations. Falling prevalence, while welcome, poses new challenges for TB surveillance. Cross-sectional disease surveys require very large sample sizes to accurately estimate disease burden, and even more participants to detect trends over time or identify high-risk areas or populations, making them prohibitively resource-intensive. In the past, tuberculin skin surveys measuring *Mycobacterium tuberculosis* (Mtb) immunoreactivity were widely used to monitor TB epidemiology in high-incidence settings, but were limited by challenges with both delivering and interpreting the test. Here we argue that the shifting epidemiology of tuberculosis, and the development of new tests for Mtb infection, make it timely and important to revisit the strategy of TB surveillance based on infection or immunoreactivity. Mtb infection surveys carry their own operational challenges and fundamental questions, for example: around survey design and frequency; which groups should be included; how the prevalence of immunoreactivity in a population should be used to estimate force of infection; how individual results should be interpreted and managed; and how surveillance can be delivered efficiently and ethically. However, if these knowledge gaps are addressed, the relative feasibility and lower costs of Mtb infection surveillance offer a powerful and affordable opportunity to better “know your TB epidemic”, understand trends, identify high-risk and underserved communities, and tailor public health responses to dynamic epidemiology.

## Background

### Tuberculosis surveillance–past and present

Almost a quarter of the world’s population has immunological evidence of prior tuberculosis (TB) infection [1]. While only a minority will develop symptomatic disease [2, 3], TB continues to kill over 1.5 million people per year [4]. TB incidence has been declining globally–although unevenly–over the past decade, but these trends are threatened by urbanisation, conflict, migration, and disruption due to COVID-19 [4–6]. In shifting contexts, National TB Programmes and the World Health Organization (WHO) need high-quality, localised, contemporary epidemiological data to guide efforts to “End TB.”

The pursuit of elimination of infectious diseases as diverse as malaria, visceral leishmaniasis and leprosy demonstrates that declining epidemics often concentrate in vulnerable populations [7–9], requiring reactive, targeted responses. Surveillance is critical to accurately estimate the burden of disease, identify high-risk geographical areas and key populations, target interventions to reduce incidence and mortality, strengthen health systems to reach those who find it difficult to access services, evaluate interventions, and track progress towards goals. Surveillance methodologies themselves also need to adapt as disease epidemiology changes.

Tuberculin skin tests (TST) have a long history of use in TB surveillance. Tuberculin, a combination of proteins derived from *Mycobacterium tuberculosis* (Mtb), was isolated by Robert Koch in 1890 and developed into a diagnostic test by Clemens von Pirquet in 1907. Intradermal injection of these antigens provokes a local reaction in people with previous mycobacterial exposure, implying Mtb infection [10]. Cross-sectional TST surveys of populations or sentinel groups such as school children and military recruits were a cornerstone of TB surveillance for much of the 20^th^ century [11]. The prevalence of tuberculin immunoreactivity was used to calculate an “annual rate of TB infection” (ARTI) [11], and to infer disease metrics using rules of thumb such as “Styblo’s rule”, which defined a fixed ratio between ARTI and disease incidence based on an estimate that each smear-positive TB case results in an average of 10 new infections per year [12, 13]. A variety of challenges with the accuracy and operability of the TST led to a shift away from this approach in the 1990s, and particularly in low-resource, high-burden settings, towards prevalence surveys of TB disease [14]. However, as we will outline below, disease prevalence surveys have their own limitations, particularly as countries target TB elimination.

The importance of ongoing TB transmission and the unmet need for high-resolution epidemiological data in many settings, combined with the development of more specific and convenient tests of Mtb immunoreactivity, should prompt policymakers to revisit the potential for surveillance based on Mtb infection. Here, we argue that well-designed immunoreactivity surveys could add considerably to understanding of global and local TB epidemiology.

### Possible targets for TB surveillance

**Fig 1:**
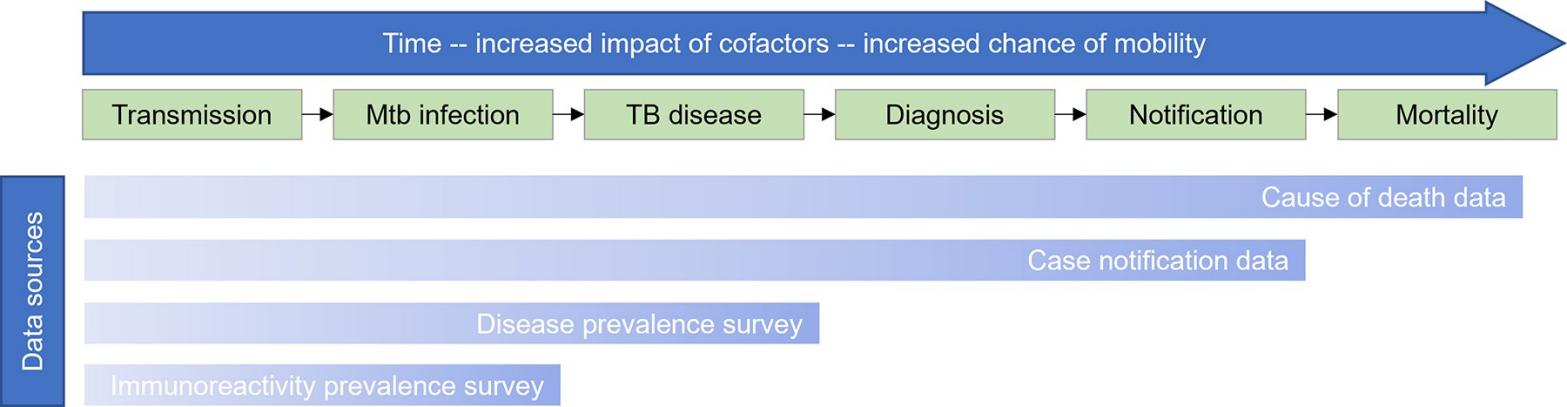
Simplified diagram of the causal stages between TB transmission, case notification and mortality. Data sources (bottom) may capture more proximal or distal stages of the process. As methods capture later stages, it becomes increasingly challenging to draw inferences about the original causative transmission event.

Classical explanations of TB pathophysiology distinguish Mtb infection from TB disease. In the former an individual has immunological evidence of *M*. *tuberculosis*, but the infection is “latent” and contained, and the person remains asymptomatic and non-infectious [15]. In TB disease this immune control is lost, and infection progresses to symptomatic, infectious illness, with tissue damage and positive microbiological tests (e.g. sputum smear, culture or Xpert) (Fig 1). This dichotomy is challenged by the growing understanding that TB is a spectrum and progression is non-linear [15–17]; nevertheless it offers a useful framework to understand the different possible targets for TB surveillance.

### Surveillance using measures of TB disease

Incidence and prevalence of disease are central measures of TB morbidity. Optimally, all people with TB disease would be diagnosed and notified centrally by public health systems, routinely delivering a representative measure of burden. However, in 2019 an estimated 30% of TB cases went unnotified, overwhelmingly in low- and middle-income countries, and this rose to over 40% in 2020 due to COVID-19 [4]. Differential access to diagnosis and registration results in systematic underestimation of the disease burden in underserved populations, which risks exacerbating existing inequities [18, 19]. Further, around 40% of TB case notifications globally are microbiologically unconfirmed, leaving the potential for routine data to be distorted by varying proportions of false positives [4].

The WHO therefore recommends cross-sectional surveys for prevalent TB disease to obtain less biased estimates in countries with high estimated TB prevalence and low case-detection ratio [20]. Since the 1990s more than 40 national prevalence surveys have been performed, leading to important insights about global TB morbidity and mortality [21], subclinical TB [22] and underdiagnosis in groups such as men and people without HIV [23–25]. However, surveys of TB disease have several critical limitations, capturing only a late stage of the TB epidemiological process (Fig 1), and often requiring very large sample sizes to do so. Mtb infection surveys may overcome some of these limitations.

## The case for revisiting Mtb infection surveys

### Measuring infection to monitor transmission

Both case notifications and prevalence surveys aim to measure TB disease, an outcome removed from TB transmission by many steps and usually by months or even years (Fig 1). While TB disease incidence and prevalence are relevant measures of burden, directly measuring TB transmission and infection may have additional benefits. TB transmission is highly heterogeneous [26, 27], with ongoing recent transmission responsible for most cases of TB in high-incidence settings [28–30]. Variations in TB transmission over short time periods (for example, due to outbreaks or COVID-19-related disruptions) or between groups or communities may be obscured if only captured at the point of disease. These limitations are compounded by high population mobility [31] and acquired risk factors for progression to disease, such as HIV or diabetes [32, 33]. Capturing transmission (for example through infection surveillance or whole genome sequencing) is therefore both a convenient, pragmatic indirect indicator of infectious disease in the population, but also an independently relevant indicator of TB epidemiology. Further, the diagnosis of infection in high-risk groups identifies individuals who may benefit from interventions to *prevent* progression, such as preventive therapy or vaccination.

### Scale and cost

The sample size (N) required to estimate prevalence of disease or infection to a designated level of relative precision is calculated using:

$$N=\frac{z^{2}(1-p)}{e^{2}p}$$

where z reflects the desired confidence level, e the relative precision, and p the prevalence. Higher sample sizes are therefore required to achieve a precise estimate at lower prevalence (S1 Fig). For example, we might select the commonly-used 95% confidence level, corresponding to z = 1.96, and a desired relative precision of 20% (e = 0.2). If the true population prevalence of a condition is 10%, a sample size of 865 is required to state with 95% confidence that the prevalence lies within the range 8–12% (10% ±20% of 10%). If the true prevalence is 1%, estimating a prevalence of 0.8–1.2% (the same relative precision) requires a sample size of 9,508; at a true prevalence of 0.1%, estimating a range of 0.08–0.12% requires a sample size of 95,924.

Critically, as the point-prevalence of undiagnosed TB disease is well below 1% in all but a few populations, disease surveys typically require tens of thousands of people to be screened with costly initial and confirmatory tests [14, 25], and still may not identify enough individuals with TB to discern high-risk groups or areas for targeted interventions (Table 1). While the relative prevalence of immunoreactivity and disease will vary, immunoreactivity is more common [1]: in five national prevalence surveys which simultaneously measured both Mtb immunoreactivity and disease, the prevalence of Mtb immunoreactivity in 5-9-year-olds was 11- to 55-times that of disease (Table 1). Accordingly, the sample sizes used by the infection surveys were 3 to 20% those of the disease prevalence surveys in the same countries, with comparable relative precision. Importantly, as surveillance targeting infection rather than disease is more affordable and efficient, it becomes feasible to achieve higher spatial resolution, and to repeat surveys to monitor trends.

**Table 1:** The ratio of prevalence of Mtb infection in children aged 5–9 years and TB disease in the population, in national surveys since 1993 in which surveillance has been performed simultaneously for infection and disease.

	TB disease prevalence survey				Mtb infection prevalence survey					Ratio of prevalence (prevalence of infection / prevalence of disease) ^3^	Ratio of survey size (infection survey size/ disease survey size)^4^
Survey	Methods	Age group (years)	Number surveyed	Number positive (smear or culture)^1^ Prevalence, % (95% CI)	TST cut-off	Age group (years)^2^	Number surveyed	Number positive Prevalence, %, (95% CI)	Calculated ARTI (95% CI)		
South Korea, 1995 [34]	Chest X-ray, sputum smear and culture if abnormal	5+	64713	142	10mm	5 to 9	5412	184	0.46% (0.39–0.53%)	15.49	0.084
				0.22%				3.40%			
								(2.92–3.88%)			
				(0.18–0.26%)							
Philippines, 1997 [35, 36]	Chest X-ray, 3 sputum smear and culture if abnormal	10+	12850	127	10mm	5 to 9	439	71	2.32% (1.80–2.87%)	16.36	0.034
				0.99%				16.17%			
				(0.82–1.16%)				(12.73–19.62%)			
Cambodia, 2002 [37]	Chest X-ray and symptom screen, sputum smear and culture if positive	10+	22160	271	10mm	5 to 9	4470	610	2.02% (1.86–2.18%)	11.16	0.20
				1.2%				13.65%			
				(1.08–1.37%)				(12.64–14.65%)			
Vietnam, 2007 [38]	Chest X-ray and symptom screen, sputum smear and culture if positive	15+	94179	269	10mm	6 to 9	8271	1052	1.69% (1.59–1.79%)	55.19	0.15
								12.72%			
				0.29%				(12.00–13.44%)			
				(0.25–0.32%)							
Bangladesh, 2009 [39, 40]	2 sputa for fluorescence microscopy, re-examined by smear. Chest X-ray if inconclusive.	15+	52098	33	8mm	5 to 9	9357	1160	1.75% (1.65–1.85%)	44.53	0.088
								12.4%			
				0.063%				(11.7–13.1%)			
				(0.042–0.085%)							

CI: confidence interval. TST: tuberculin skin test. ARTI: annual risk of TB infection.

^1^Disease prevalence estimates refer to microbiologically-confirmed TB, here defined as any positive smear or culture result; prevalences are presented as simple percentages.

^2^Several studies included additional age groups, but results from 5- or 6-to-9 year-olds included here for consistent comparison.

^3^Ratio of prevalence is calculated by dividing the prevalence of Mtb infection in the infection survey, with the prevalence of TB disease in the associated disease survey.

^4^Ratio of survey size is calculated by dividing the number of participants recruited in the infection survey with the number recruited in the disease survey. This refers to the actual number of participants surveyed, not the theoretical number required to achieve a set precision; the relative precisions of the ARTI and the disease and infection prevalence are demonstrated by the estimate CIs, and are generally comparable within and between studies.

### Diagnostic limitations and advances

All surveillance methodologies face a tension between sensitivity, specificity and costs. TB disease prevalence diagnostic algorithms may include symptom screening (which is insensitive, non-specific and may be affected by differences in symptom perception [20]), and more costly tests including chest radiography (moderately sensitive with low specificity), and microbiological tests (specific but insensitive). Depending on the methodology used, those with subclinical disease, smear-negative disease, minimal X-ray changes or extra-pulmonary TB may be missed. These limitations are particularly marked in some groups: for example, paediatric TB is challenging to diagnose and is therefore generally excluded from disease prevalence surveys [14], limiting our understanding of epidemiology in children.

Tests for Mtb “infection” are assays of immunological memory of Mtb, and include *in vivo* responses to intradermal injection of mycobacterial antigen preparations (such as tuberculin), or *in vitro* interferon-gamma release assays (IGRAs). These are only a proxy for true, viable infection: immune memory of Mtb may be seen in people with very distant exposure at low risk of reactivation [16], has a poor individual-level predictive value for future development of disease [2, 3], and may correlate poorly with novel methods aimed at detecting viable infection [41]. However, individual-level precision is less important when infection tests are used to detect an epidemiological signal at a population level. Tests of Mtb immunoreactivity correlate with the TB exposure experienced by individuals and populations [42, 43], and both the prevalence and incidence of immunoreactivity correlate, albeit imperfectly, with the prevalence of disease [43, 44].

Recent diagnostic developments may overcome some critical limitations in Mtb infection surveillance [45]. For example, TST’s cross-reactivity with BCG vaccination and environmental non-tuberculous mycobacteria results in nonspecific positivity which becomes more salient as incidence of true *M*. *tuberculosis* infection falls [12, 46]. IGRAs use *M*. *tuberculosis*-specific antigens, but their cost and need for phlebotomy, overnight incubation and laboratory processing have made them challenging to deploy in large-scale surveillance, especially in low-resource settings [47]. Newer advances include specific skin tests such as C-Tb, Diaskintest and C-TST, recommended for use by WHO in 2022 [48]; these use the same *M*. *tuberculosis*-specific antigens (such as ESAT6 and CFP10) as IGRAs, but in the skin test formulation which avoids the need for laboratory processing [49–51]. Secondly, novel IGRA assays reduce the need for laboratory processing by using alternatives to enzyme-linked immunosorbent assays to measure interferon-gamma production (e.g. the QIAreach-QFT semi-automated lateral flow immunoassay) [52, 53]. Novel assays aiming to measure recency of infection, persistence or risk of progression are not currently practical for large-scale screening [54–57].

## Considerations for Mtb infection surveys

Disease prevalence surveys will only become more challenging as TB prevalence falls. Coupled with the new diagnostic opportunities, this should prompt a re-evaluation of a potential role for surveys of Mtb immunoreactivity in surveillance. If Mtb infection surveys are to be more widely adopted, there are several important considerations in how they are implemented and interpreted, which will materially affect their usefulness. Below, we outline some of these considerations (Table 2), and highlight areas where more research is required.

**Table 2:** Core considerations for surveillance based on Mtb immunoreactivity.

1. How should Mtb immunoreactivity prevalence be interpreted? 2. Which age groups should be tested? 3. How should serial surveys be performed? 4. Which diagnostic tests should be used, and how? 5. What are the practical and operational considerations? 6. How should those with positive tests be managed? 7. Is Mtb infection surveillance affordable? 8. Is Mtb infection surveillance ethical and acceptable?

### How should Mtb immunoreactivity prevalence be interpreted?

Infection surveys may capture either the prevalence of Mtb immunoreactivity (reflecting participants’ cumulative incidence of Mtb infection), or, in serial surveys, the period incidence of infection in individuals or populations. However, while the prevalence of immunoreactivity *per se* is intuitively interpretable, it captures an asymptomatic state of exposure rather than a disease state or population “burden”, and is therefore of less inherent epidemiological interest than the population force of infection, which implies ongoing transmission from infectious individuals. For this reason, prevalence of TST- or IGRA-positivity has traditionally been used to estimate the ARTI, incorporating mean age to account for the number of years of exposure using the formula:

ARTI=1−(1−Prevalence)1Age

**Fig 2:**
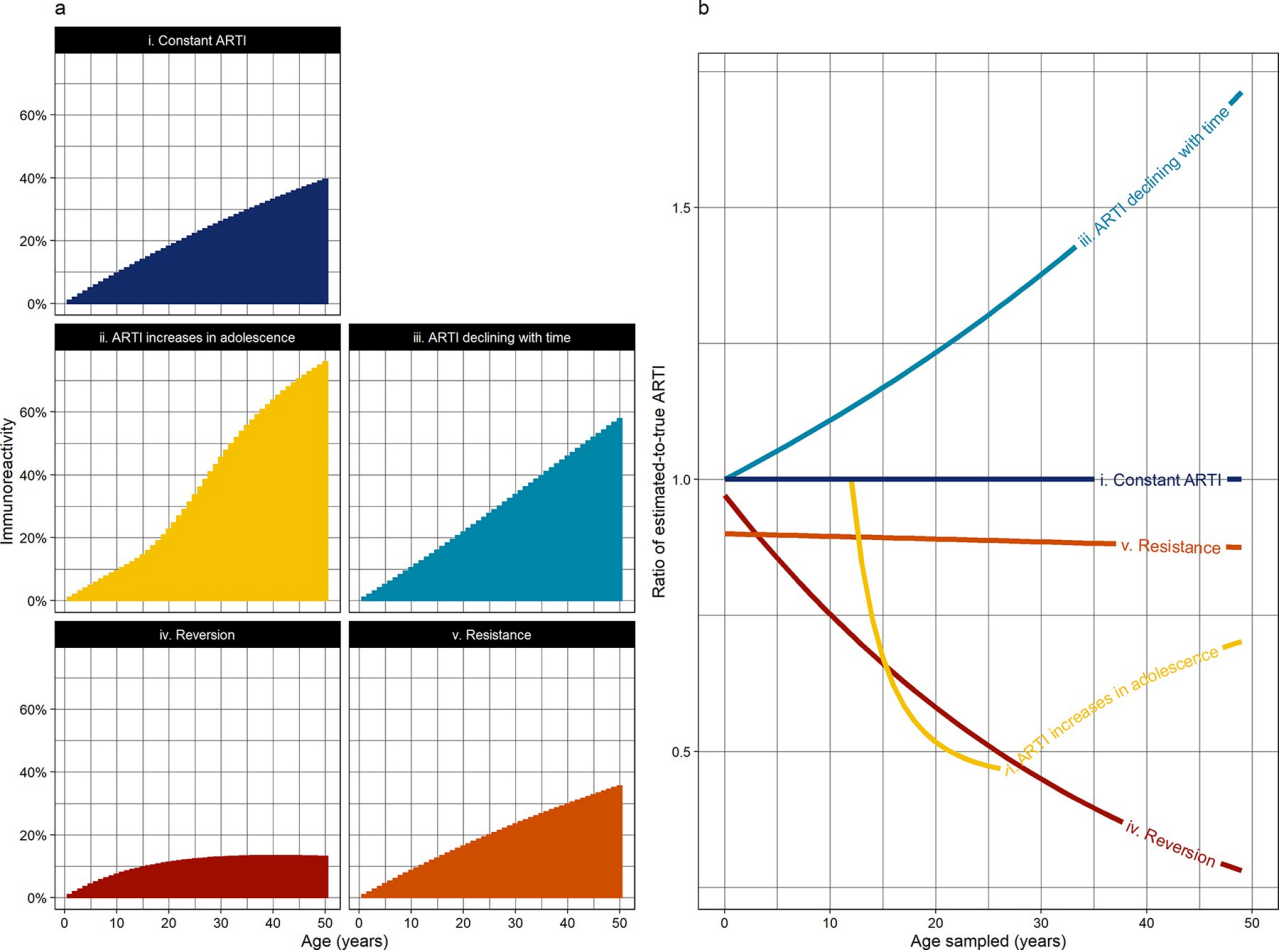
a. Age-dependent cross-sectional population prevalence of Mycobacterium tuberculosis (Mtb) immunoreactivity under differing epidemiological assumptions: i) Constant annual risk of tuberculosis infection (ARTI) of 1%, with no reversion, secular trends or age-related trends; ii) Childhood ARTI of 1% which increases from age 12 to a maximum of 4% in adulthood [58]; iii) Current ARTI of 1% in all age groups on a background of a secular decline in ARTI of 2% per year[4]; iv) Constant ARTI of 1%, but with 5% annual reversion [59]; v) Constant ARTI of 1%, assuming 10% of the population are resistant to infection and remain immune-nonreactive [60]. b. The degree to which age-specific ARTIs would be under- or over-estimated in each scenario, by age group sampled, if a constant rate of infection with no reversion were assumed (i.e. using **ARTI = 1−(1−Prevalence)** ^**1/Mean age**^).

However, this formula relies on several unsafe assumptions, which may lead to inaccurate estimation of the true ARTI (Fig 2).

The first assumption is that the force of Mtb infection is constant with secular calendar time. In reality, transmission has been steadily declining in most parts of the world due to improved living standards, antiretroviral therapy scale-up, and increased access to diagnosis and treatment [4]. Threats to this trend include urbanisation, displacement, and more recently the COVID-19 pandemic, which has profoundly disrupted multiple epidemiological indicators and covariates [4, 6]. As such, most individuals’ lifetimes encompass periods of varying TB exposure.

The second assumption is that force of infection is constant with age [61]. Social contact studies, however, show pronounced change in respiratory contact networks from adolescence, with age- and sex-assortative mixing causing young adults, particularly men, to have increased contact with potentially infectious individuals [23, 58]. This is consistent with Mtb infection surveys which show increased ARTI during adolescence and young adulthood [62–64], again most marked for men [62].

The third assumption is that immunological tests reliably become positive when a person is infected, and then remain positive for life. Again, we know that these are oversimplifications [61]. Reversion of results from positive to negative is well-documented [59, 65, 66] and may vary by age [66]. A minority of individuals also appear to demonstrate “resistance”, remaining negative on immunological tests despite intense exposure to infectious TB [60, 67, 68]. Failure to account for either effect can severely underestimate the true transmission rate in the population [61, 69], and these discrepancies are compounded when older people are surveyed (Fig 2).

It is likely that *all* the effects illustrated in Fig 2 occur simultaneously, with relative magnitudes that are unknown and vary between populations. If immunoreactivity surveys are deployed, more nuanced methods must be used to estimate infection incidence from infection prevalence in different age groups, which incorporate these variables and their associated uncertainties. These methodologies should be consistent to allow tracking of trends over time, comparison of populations and evaluation of interventions, at a defined level of precision. Standardised methodologies and reporting frameworks should be used, to support collation by international bodies and integration with other data sources.

A separate consideration is whether this estimated force of infection should be tracked as an independent metric of TB epidemiology, or whether it can also be used to infer other measures of TB burden, such as incidence of disease and mortality rate (the focus of current EndTB targets). Again, the relationship between these metrics has been oversimplified in the past, using “rules of thumb” such as Styblo’s rule which have proved inaccurate in the contemporary era [43, 70] (Table 1), and more nuanced approaches are required if infection incidence is to be used as a proxy for disease.

### Which age group(s) should be tested?

Given that the force of Mtb infection varies with age and secular time, the choice of which age group(s) to survey can profoundly impact results and their interpretation. Immunological evidence of Mtb infection reflects cumulative lifetime TB exposure, which in young children (e.g. under-5s) is by definition recent exposure. Immunoreactivity in young children provides a useful guide to contemporary ARTI, whereas as age increases, prevalence can be increasingly dominated by historic exposure and impacted by reversion (Fig 2). Additionally, young children are generally less mobile, making them potential sentinels of recent local transmission [55].

One disadvantage to restricting surveillance to young children is their lower prevalence, due to both their shorter cumulative risk and likely higher force of infection during adolescence and early adulthood [58, 71]; larger samples of young children are therefore required to identify enough positive cases to achieve relative precision and resolution. Moreover, while Mtb infection rates measured in young children can be usefully compared between different communities, they likely underestimate the force of infection in adults, and may preferentially capture household transmission, while we know that most cases of TB disease arise from transmission outside the household [72, 73].

The optimal group, or combination of groups, to survey may vary between populations, epidemiological situations and key outcome of interest. However, standardised approaches are also required to allow consistent interpretation and comparison.

### How should serial surveys be performed?

A single survey inevitably captures only a snapshot of infection risk. Serial surveys are required to understand changes, and to resolve age-related and secular trends in TB transmission. This can be achieved by testing a repeat representative cohort of the same population (for example school children or military recruits) [74]. An alternative is serial testing in the same *individuals*. This has the advantage of being able to confirm whether an individual has converted from negative to positive, suggesting recent infection amenable to preventive therapy and contact-tracing, and may also reveal the incidence of test reversion. However, it requires additional resources to trace individuals, may be of uncertain acceptability, and in the case of injectable tests may give false-positive reactions due to sensitisation from previous antigen injection [75, 76]. Each round of sampling requires additional resources, and the optimal interval between surveys has not been clearly defined. In part this will depend on the intended purpose of the survey (for example, comparison between populations or tracking trends over time), and the anticipated magnitude of change or difference which is felt to be of public health significance.

### Which diagnostic tests should be used, and how?

The lack of a gold-standard test for Mtb infection makes it challenging to assess the performance of novel diagnostics. Specific skin tests (such as C-Tb, Diaskintest and C-TST) have sensitivities of 75–91% in people with TB disease, high specificity amongst those at low risk of TB, and an agreement of 80–87% with IGRA [48, 51]. Newer IGRA platforms, such as the QIAreach-QFT, appear promising but have not been evaluated in population-based studies [53]. There have been no direct head-to-head comparisons between the newer skin tests and newer interferon-gamma release assays, either with respect to their diagnostic performance or their feasibility and acceptability in practice.

Mtb infection tests are interpreted by converting a continuous immunological measure (interferon-gamma release or skin induration) into a binary positive/negative result. There is incomplete consensus on the most appropriate thresholds [2] (Table 1) or whether the same thresholds are appropriate for capturing individual risk (influenced by age, HIV status and other factors) *vs* population exposure. Quantitative IGRA responses are associated with the intensity of recent TB exposure [77] and with risk of progression to active TB [78], but further research is needed to optimise inclusion of continuous distributions into models of Mtb infection burden and transmission and disease.

In systematic reviews, TST and IGRA both have a positive predictive value well below 5% for detecting people who will develop TB disease over two years [2, 3], and while the necessary longitudinal data is not available, we assume that the newer assays will share the same limitations. Further, more research is required on these assays’ reversion rates, which may strongly impact epidemiological interpretation [69].

Both IGRAs and skin tests have notable barriers to scale-up. While skin tests do not require laboratories, reagents do need to be maintained in a cold chain with limited shelf-life once opened. Both placement and interpretation require training and quality-control, and the need for a second visit which increases burden and loss-to-follow-up [46, 79]. Self-reading or mHealth (using smartphones to assess induration) are possible alternatives [80–82], although these may not be appropriate for all settings. IGRAs require phlebotomy, which may be problematic in young children (although it would permit combination with screening or surveillance for other conditions), and the requirement for incubation means they still cannot give same-day results [52, 53].

### What are the practical and operational considerations?

Operational decisions about where and how to perform testing will often be dictated by the age groups recruited and the testing modality chosen. Historical tuberculin surveys often took place in easy-to-locate school cohorts; however this restricts sampling to school-age children, and may be systematically biased by school attendance [12]. Disease prevalence surveys often rely on household-level sampling which is more intensive, and tends to under-sample working-age men and more vulnerable, high-mobility populations [25]. Care is therefore needed to reach the highest-risk individuals, to accurately estimate burden and promote equity of access to care.

Surveillance may be most sustainable when integrated within existing systems, rather than in vertical single-disease siloes. For example, young children could be tested when they attend primary health settings for routine vaccinations, or in combination with demographic health surveys or surveillance for other infections, such as malaria or neglected tropical diseases [83, 84]. Useful data may also be obtained from sentinel populations, such as antenatal clinic attendees, students, or healthcare workers, although these will require adjustment to generate population-representative estimates.

### How should those with positive tests be managed?

It is important to distinguish between *screening* for Mtb infection (with the expectation of individual treatment) and *surveillance* using immunoreactivity as a marker of exposure (the focus of this article). Regardless, resources and infrastructure must be in place to appropriately manage people with a positive test. This should include an assessment (with symptom screen and/or diagnostic tests) for TB disease, and onward referral as required. The optimal screening algorithm in low-risk, asymptomatic community members with a positive Mtb infection test has not been clearly defined.

Once TB disease has been “excluded”, preventive therapy should be strongly considered for defined high-risk groups (e.g. young children, recently exposed and people living with HIV) [85–87], meaning that immunoreactivity testing can be combined with a potentially beneficial preventive intervention. Newer three- or one-month rifapentine-based regimes make preventive therapy more attractive and feasible for both patients and providers [88–90].

In other groups, the risk-benefit balance is complex. The high prevalence of Mtb immunoreactivity (much of which, in the setting of falling incidence, may represent historic exposure) [1], the low probability of progression to TB disease [2, 3], the risk of reinfection in high-prevalence settings, and the potential toxicities of preventive therapy, mean that it is neither practical nor desirable to offer therapy to all those with positive infection tests. Evidence-based tools may inform individualised decision-making [91], but are hard to deploy in a public health approach. Furthermore, it can be challenging to link those who *are* eligible for treatment to appropriate therapy, and to promote adherence in asymptomatic people who perceive their risk to be low [92, 93]. Improved strategies are required to better predict those who are most likely to benefit from preventive therapy, or newer interventions such as vaccination.

### Is Mtb infection surveillance affordable?

The costs of TB disease prevalence surveys have been estimated at $4–15 per person surveyed, but this will be higher if chest radiography is used, and/or if microbiological testing is performed for everyone regardless of symptoms [14]. Mtb infection surveys require much lower sample sizes (Table 1), and while their costs will also vary according to the diagnostics used and the initial investments in equipment and infrastructure required, they are likely to be considerably less expensive. Strategies may be more affordable if integrated within current surveillance systems or performed in conjunction with other programmatic activities, allowing costs to be shared and initial start-up costs to be reduced.

### Is Mtb infection surveillance ethical and acceptable?

While there is a positive moral obligation to deliver public health surveillance [94, 95], Mtb immunoreactivity surveys entail several ethical issues [96, 97]. They require the burden of either phlebotomy from, or administration of skin tests to, otherwise healthy individuals for the purposes of public health. Participants may be harmed by a stigmatising label of “TB infection”, and the potential toxicity of preventive therapy for uncertain benefit [98].

Tuberculin surveys have used globally and are assumed to be acceptable. However, searching OVID MedLine with terms relating to Mtb infection and qualitative research or acceptability revealed no articles exploring the views of communities on population-level surveillance with either IGRA or TST. The fact that vertically-administered programmes have previously been tolerated should not be taken as evidence of endorsement. For example, skin-snipping is an established but invasive method for onchocerciasis surveillance, which has been met with increasing community rejection [84]. Suspicion around vaccinations against TB and COVID-19 in many communities demonstrates how an injectable test could understandably provoke mistrust [99–101]. Given these ethical complexities, informed consent is essential, requiring time and culturally-appropriate communication to explain the concept of “immunoreactivity”, the purpose of testing, and the implications of a positive result for a potentially communicable and stigmatised infection [96, 102, 103]. This is particularly critical because TB disproportionately affects underserved members of society, who may be especially vulnerable.

If effective, TB surveillance should facilitate pro-equity approaches to targeted prevention, by identifying communities which may benefit from prioritised case-finding, disease management, infection prevention and/or HIV services, as well as interventions to address overcrowding and poverty. However, if the methodology is not robust and groups are missed or excluded, it may only divert resources from universal healthcare, and worsen pre-existing injustice.

### Recommendations

Dynamic epidemics require adaptive surveillance methodologies, and population-wide surveys for undiagnosed TB disease become increasingly problematic as prevalence falls. While in the longer term there is a clear need for diagnostics which more accurately capture relevant properties of Mtb infection, such as recency and persistence, existing immunoreactivity tests can provide valuable information at a population level.

Maximising the insights from Mtb immunoreactivity surveillance requires further research to address the knowledge gaps we have highlighted (Table 3). Informed by this research, we advocate that WHO support development of recommendations to define the respective roles of infection disease prevalence surveys in TB surveillance. Finally, if immunoreactivity surveys are to be more widely used, consensus methodologies for how to conduct them must be developed and updated to reflect new knowledge [11]. They should encompass standardised operational guidelines (for example around recommended diagnostics, cut-offs and age-groups to recruit), definitions of outcome parameters of interest, and reporting frameworks which facilitate collation, comparison and integration with other data sources.

**Table 3:** Research gaps, existing evidence and priority research methods to inform wider implementation of Mtb infection surveillance.

Key questions	Existing evidence	Priority research methods
How does ARTI vary by age and sex? How does this impact the interpretation of Mtb immunoreactivity prevalence?	Social contact data suggest force of infection increases in adolescents and males [58]. This is reflected in cross-sectional surveys [62–64], but has not been systematically examined across age-groups and populations.	Synthesis and meta-analysis of available data on age- and sex-specific prevalence and incidence of Mtb immunoreactivity in different epidemiological settings.
What are the performance characteristics of newer TB skin-tests and IGRAs in population settings? What causes discordance with reference tests?	A WHO evaluation estimated that novel TB skin tests had pooled sensitivity of 76% and specificity of 98% against IGRA and TST [48]. Small studies of QIAreach-QFT have shown high (kappa = 0.96) agreement with QFT-Plus, and moderate (kappa = 0.42) agreement with TST, with a 2022 WHO policy statement recommending further evaluation of the assay’s reproducibility and predictive accuracy [104].	Adequately powered population-based evaluations of newer skin tests and IGRAs, including comparison with existing reference tests and long-term follow-up for development of incident disease. Further evaluation in children and people living with HIV.
What are the appropriate cut-offs for tests of Mtb infection when used at population level? Should continuous measures be used, and if so how should they be analysed to estimate force of infection and included in burden estimation models?	Quantitative IGRA responses are associated with intensity of recent exposure [105], and with risk of progression to active TB [78]. Cut-offs for novel tests have been defined in small-scale evaluations, and largely in reference to existing benchmarks (TST or IGRA) [49, 50, 106]. The significance of the quantitative result of new tests has not been fully evaluated.	Population-based evaluations of specific skin tests and IGRAs, which include quantitative measurement and long-term follow-up for development of incident disease.
How does reversion of the novel Mtb infection tests impact the interpretation of Mtb surveys?	Reversion of TST and IGRA is well-described [59, 65, 66], and may significantly impact the interpretation of Mtb infection surveys [61, 69]. Reversion has not been fully explored for novel tests.	Serial evaluations with specific skin tests and IGRAs to investigate reproducibility and reversion, both spontaneous and following preventive therapy. Accurate estimates of the rates of reversion to allow modelling of its impact on population estimates.
Do Mtb infection surveys provide a feasible, affordable alternative or complement to disease prevalence surveys?	Tuberculin surveys have been used for decades, albeit with operational challenges [11, 12]. Diaskintest has been used for surveillance in Russia for years [107]. Because of cost and laboratory requirements, most epidemiological surveys using IGRAs have been in the context of trials rather than routine community surveillance.	Pilot immunoreactivity surveys using novel diagnostics, in research or as part of new National TB disease prevalence surveys, alongside implementation and cost-effectiveness research. Explore optimised sampling approaches to maximise efficiency.
Are epidemiological surveys of Mtb infection acceptable?	Skin test surveys have been used for decades, but there is limited research exploring their acceptability.	Qualitative and mixed-methods research nested within population-based infection surveys, exploring perceptions of surveillance based on exposure rather than disease, and the relative acceptability of different diagnostics.

## Conclusion

Surveillance efforts must overcome the paradox of TB’s low point-prevalence of disease in most populations, but its huge mortality and morbidity. Integrating Mtb infection measurement into global TB surveillance may offer a feasible, affordable way to track trends and target efforts towards underserved populations. We have highlighted research questions which need to be answered in light of our evolving understanding of TB transmission, pathology and epidemiology, to better understand the performance of novel diagnostic tests and how their results should be interpreted, in order to fully realise the potential of this methodology.

## Supporting information

S1 Fig Impact of prevalence on the sample size required to achieve 95% confidence in an estimate at different levels of relative precision. The upper panel encompasses the range of prevalence of Mtb infection commonly observed in high-prevalence settings, while the lower panel zooms on the usual range of prevalence of TB disease.
(TIF)
