# Supplementary figures and images for: Know your tuberculosis epidemic–Is it time to add Mycobacterium tuberculosis immunoreactivity back into global surveillance?
Source: PLOS Glob Public Health. 2022 Oct 24;2(10):e0001208. doi: 10.1371/journal.pgph.0001208 (PMC10021854; doi:10.1371/journal.pgph.0001208)

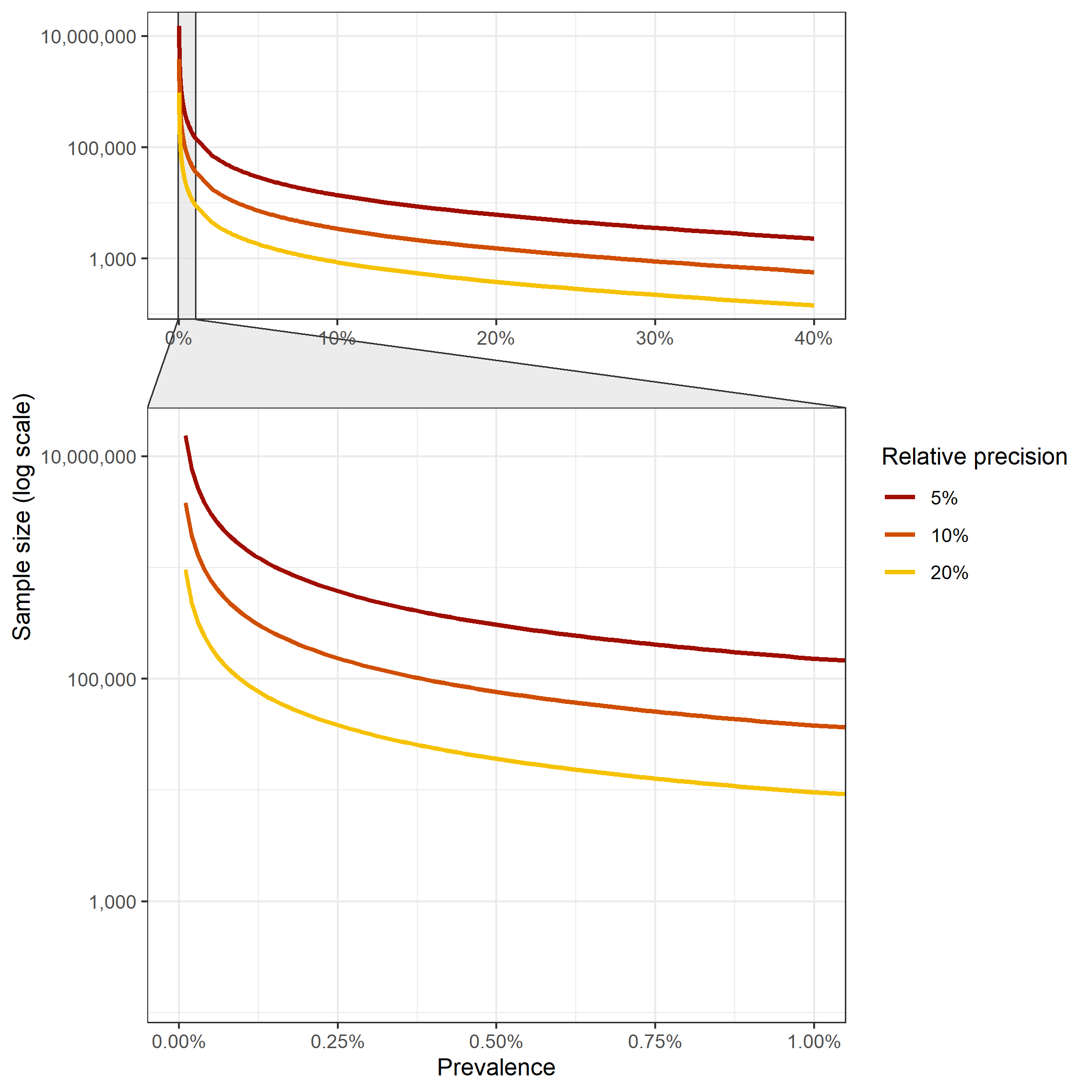

Supplement: S1 Fig — The upper panel encompasses the range of prevalence of Mtb infection commonly observed in high-prevalence settings, while the lower panel zooms on the usual range of prevalence of TB disease. (TIF) [file pgph.0001208.s001.tif]
